# Ethical considerations in design and implementation of home-based smart care for dementia

**DOI:** 10.1177/09697330211062980

**Published:** 2022-02-01

**Authors:** Christine Hine, Ramin Nilforooshan, Payam Barnaghi

**Affiliations:** Department of Sociology, University of Surrey, Guildford, UK; Surrey and Borders Partnership NHS Trust & University of Surrey, Guildford, UK; Department of Brain Sciences, UK Dementia Research Institute (UK DRI), Imperial College London & Care Research and Technology Centre, London, UK

**Keywords:** Smart care, artificial intelligence, machine learning, dementia, autonomy, beneficence, non-maleficence, explicability, fairness

## Abstract

It has now become a realistic prospect for smart care to be provided at home for those living with long-term conditions such as dementia. In the contemporary smart care scenario, homes are fitted with an array of sensors for remote monitoring providing data that feed into intelligent systems developed to highlight concerning patterns of behaviour or physiological measurements and to alert healthcare professionals to the need for action. This paper explores some ethical issues that may arise within such smart care systems, focusing on the extent to which ethical issues can be addressed at the system design stage. Artificial intelligence has been widely portrayed as an ethically risky technology, posing challenges for privacy and human autonomy and with the potential to introduce and exacerbate bias and inequality. While broad principles for ethical artificial intelligence have become established, the mechanisms for governing ethical artificial intelligence are still evolving. In healthcare settings the implementation of smart technologies falls within the existing frameworks for ethical review and governance. Feeding into this ethical review there are many practical steps that designers can take to build ethical considerations into the technology. After exploring the pre-emptive steps that can be taken in design and governance to provide for an ethical smart care system, the paper reviews the potential for further ethical challenges to arise within the everyday implementation of smart care systems in the context of dementia, despite the best efforts of all concerned to pre-empt them. The paper concludes with an exploration of the dilemmas that may thus face healthcare professionals involved in implementing this kind of smart care and with a call for further research to explore ethical dimensions of smart care both in terms of general principles and lived experience.

## Introduction

This paper explores ethical challenges pervading design and implementation of a potentially transformative new technology for healthcare. Artificial intelligence (AI) offers the ability to build computer systems that can learn patterns from a training data set and apply those patterns to new data^1^ with the prospect of enhancing decision-making through identification of patterns that humans alone cannot visualize and the ability to operate on data at a scale far beyond human capacity. The advent of digital communications and Internet-connected digital devices provide for the collection of the massive quantities of data that fuel such systems.^1^ AI has become a significant field of technology development in recent years, with the prospect of transforming decision-making in public service and in commercial settings, albeit with considerable risks and ethical challenges associated.^2^ Within this broader context of developments in machine learning an array of prospects for transformations in healthcare have been envisaged including the potential to inform and enhance clinical decision-making, helping in the provision of accurate and timely diagnosis and targeted interventions through the ability to spot patterns of interest in large amounts of data.^3^

With such visions in mind, it is a core aspiration of the UK’s National Health Service long-term plan^4^ that ‘digitally enabled care will go mainstream across the NHS’. This vision focuses on delivering personalized care in an efficient manner, freeing up healthcare professionals for targeted interventions and allowing service users to take more responsibility for their own health. AI is a core component of this vision, with a view to supporting decision-making through analysis of patient data, enabling automated alerts to direct clinical attention to the most urgent sites for intervention. Data are envisaged as being delivered through sensors and wearable devices and through data captured as a by-product of medical care. The NHS has made considerable investment in the development of AI, most notably in the establishment of the NHS AI Lab (https://www.nhsx.nhs.uk/ai-lab/) in 2019. While there are major challenges in integrating AI into clinical pathways and a risk of hype exceeding realized benefits for some time to come,^3^ there is realistic prospect of widespread usage on the horizon.

Within the field of care for long-term conditions and for an ageing population in particular there is strong motivation to provide systems that allow patients to remain at home while being monitored remotely, and such work has a considerable history.^5^ Remote monitoring holds promises for improved outcomes and increased efficiency of care in a highly resource intensive sector. The cost of care for people with long-term conditions is up to 70% of total health and social care expenditure and accounts for 50% of all GP appointments.^6^ Hospitalisation rates for people living at home with dementia are high and reduction in emergency admissions is a key priority.^7^ In the field of dementia care, AI solutions can allow both informal carers and healthcare professionals to receive alerts about troubling patterns of activity in vulnerable older people and those living with dementia in their own homes with a view to providing timely support. Recent experiences with the COVID-19 pandemic have considerably heightened the interest in providing care ‘in place’ and reducing the need for frequent or routine contact with healthcare professionals.^8,9^ In the UK within the NHS, a system of remote monitoring and smart care for people living with dementia, called Technology Integrated Healthcare Management (TIHM), was recently developed by Surrey and Borders Partnership NHS Foundation Trust in conjunction with University of Surrey and Howz commercial providers of remote monitoring systems.^10–12^ The initial trial system then formed the basis for a remote monitoring service extending to over 600 people living with dementia or mild cognitive impairment during the COVID-19 pandemic.

The TIHM system provides a case study to explore ethical issues for smart care in the specific context of people living with dementia within their own homes. Many of the issues explored here generalize to the opportunities and challenges of smart care across a range of conditions, but there are some specificities to the implementation of smart care for dementia due to the distinctive medical, psychological and social aspects of the condition and these shape the practical and ethical challenges that researchers and developers face. Dementia often involves fluctuating decision-making capacity^13^ and this poses challenges in respecting autonomy and protecting people’s interests when their capacity to make decisions is diminished. It is also important to take a flexible approach to implementation of smart care that recognizes the needs and perspectives of participants living with dementia within ecologies of care that involve both formal and informal carers^14^ and also to maintain the sense of security and refuge that people associate with home^15^ and to take dignity^16^ in care into account. These aspects of the experience of dementia add complexities to the notions of personalized care and patient choice that pervade high-level discussions of the rationale for smart care, such as the NHS long-term plan^4^ and prompt careful attention to design and delivery of appropriate and ethical smart care.

The aim of this paper is to review the current state of affairs in smart care with particular attention to ethical aspects of such technologies as applied within the context of dementia care in the home. In doing so, the intention is to outline steps that may be taken before a system reaches the implementation stage in order to build in ethical principles. Further to this, the paper highlights a set of potential ethical issues that may be more difficult to anticipate and to mitigate through system design alone. These additional ethical issues emerge from the lived experience of people living with dementia and their formal and informal carers. As smart care becomes more widespread many healthcare professionals may need awareness of these issues, to support a move to new ways of working. The intention of this paper is to assist by fostering the sensitivities to underpin an ethics of practice for those involved in smart care.

### Ethics of AI in smart care design

There has been considerable discussion of the potential for AI to pose ethical risks.^17,18^ While AI has great potential for societal benefits it also brings risks of insidious but consequential negative impacts and we therefore need to develop frameworks to anticipate and mitigate these risks. A set of general principles for ethical AI offer guidance on the issues such frameworks should attend to^17^:

• Beneficence: AI should be implemented in the interests of humanity, serving the common good.
• Non-maleficence: AI should be designed to avoid harms and in particular infringements on personal privacy and security
• Autonomy: ability to take and implement decisions should not be entirely handed over to automated systems and human input or oversight should be retained.
• Fairness: there is a risk when implementing AI systems that forms of bias and discrimination may inadvertently be built into systems and thus an overt ethic of fairness or justice toward those affected by the system should be followed
• Explicability: AI systems may be highly complex and opaque, but there is an ethical requirement for accountability and transparency in order to facilitate trust that the other ethical principles of beneficence, non-maleficence, autonomy and fairness are met.

While there is considerable consensus that these ethical principles are desirable, there is as yet no coherent mechanism for achieving conformity.^19^ Outside of the ethical review systems provided in specific contexts such as healthcare or university research settings, there is no widely applied institutional framework for delivery of ethical governance of AI.

Healthcare presents a domain in which the potential gains from AI are particularly apparent and yet the ethical risks of unfair or undesirable outcomes at levels from individual to societal are also stark.^20,21^ Any bias embedded in decision tools can have consequences for equal access to healthcare. For example, where the data used for training the system do not reflect the full population, the system may operate less well to serve the needs of already marginalized social and ethnic groups. Such bias may be difficult to question if there is high trust in automated decisions and the complexity and opacity of systems may make it difficult to understand just what the basis for the decision was and to recognize bias when it happens. There are also issues to consider in the collection of data and in the design of services to implement interventions that are informed by AI. As Mittelstadt’s^22^ review shows, the burgeoning of remote monitoring has many potential benefits in healthcare, but there are significant risks occasioned by the sensitivity of the data. The ethical status of these systems is often not clearcut: for example, Mittelstadt^22^ highlights the potential privacy tradeoffs of a smart home monitoring system that may pose some intrusion but also preserve autonomy by helping someone to stay out of residential care for longer than they might otherwise do safely.

Off-setting this array of ethical risks is the well-established practice of ethics in medical research and healthcare settings. Indeed, the ethical principles established for AI (beneficence, non-maleficence, autonomy and fairness) are very recognizable from a medical setting and would be expected to form the basis for treatment decisions whether or not AI might be involved.^23^ The principles of informed consent for participation in research and for treatment to be undertaken within the clinician/patient implied contract remain in place regardless of the technology involved. However, these may become complex to apply in the context of fluctuating decision-making capacity attendant on dementia^13^ and dilemmas may arise as clinicians may find that their understandings of ethical care and the role they play within delivery of care are shifted by the active role played by AI. Any introduction of AI to a clinical pathway would need to be justified in terms of existing medical knowledge and systems would often be subject to review as a medical device. Even with such safeguards, however, systems may not perform perfectly and there will be a need to discuss where responsibility lies when things go wrong and whether the potential fallibility of intelligent systems needs to be explained to those receiving care. Within healthcare, innovations drawing on AI are subject to the standard forms of ethical governance applied to research and innovation in that context. Such pre-emptive review aims to identify and mitigate any potential ethical harm that may be apparent at that stage. Reviewers may not, however, have a specific expertise in the design of AI such that they can evaluate the ethical consequences of technical design and take into account impacts across the lifetime of a system and in relation to individuals, specific social groups and wider society.^24^ To that end, Morley and Floridi^24^ argue that a standardized set of guidelines for ethical review in this territory is still much needed. In the absence of such guidelines, the ability of AI in healthcare to conform to ethical principles is thus heavily reliant on the ethical sensitivities of developers and their ability to translate broad ethical principles into specific design choices. In the next section, some ethically significant aspects of design in smart healthcare are explored drawing on examples from the TIHM system.

### Designing ethics into smart healthcare

**Figure 1.**
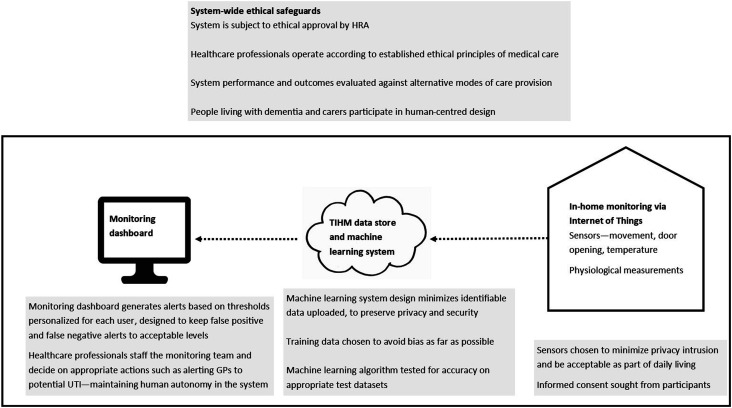
Ethical considerations associated with the design of the TIHM remote monitoring system.

System development inevitably happens as a multi-disciplinary exercise. Healthcare professional involvement is required both at the leadership level to champion projects and on an everyday basis to provide the expertise and input into the operation of trial systems. Patient involvement in development is increasingly an expectation within a healthcare environment. On the technical side, disciplinary expertise in machine learning, data science and engineering is often located within academic research environments although commercial involvement is also common in this field. This multi-disciplinary endeavour generally requires ethical review within the medical context but for trials without patient involvement university-based ethical review may be conducted instead. In either case, the starting point for ethical review will be the team’s presentation of the steps that they have taken to anticipate and mitigate ethical harms. The multi-disciplinary environment enables input into the design process from a range of different forms of ethical expertise, supporting design decisions that incorporate both the traditions of ethical healthcare and university-based researchers together with lay views.^25^ Running studies in the healthcare system also requires ongoing meetings with information technology and information governance departments in the NHS, with each group needing to understand the remit and redlines of the other groups. This array of concerns often translates into decisions about specific aspects of the system design and implementation, as summarized in Figure 1. In the remainder of this section, we describe in more detail how the key decisions depicted in Figure 1 orient to ethical principles, broadly construed.

### Human-centred design

In line with the broad principles of ethical AI and healthcare, it is important to build an infrastructure that preserves autonomy and human judgment, enabling healthcare professionals to monitor alerts and draw on established clinical pathways for action ‘using monitoring as a way to initiate rather than replace dialogue between patient and doctor’.^22^ This approach was taken in the TIHM system, developing a monitoring dashboard for use by a clinical monitoring team who assess alerts and take actions according to pre-determined clinical pathways. For example, an alert that a patient was at risk of urine infection would result in contact to suggest a visit to their GP, rather than automatically leading to prescription of treatment.^26,27^ The dashboard ranks alerts according to potential seriousness and differentiates technical, clinical and environmental alerts, with a view to directing issues to the appropriate team members and informing rather than replacing clinical judgment. From the outset a co-design approach has been taken, recruiting users to a trusted user panel who inform ongoing development.^28^ In the context of systems aimed at supporting those living with dementia, the term ‘users’ tends to include both patients and carers as interested parties. It should be noted, however, that such involvement in itself represents a burden, and there is limited potential for users to inform the highly technical aspects of machine learning design that require expert assessment of the implications and viability of various design aspects of the system with implications for usability, accessibility, compliance and privacy and ethical concerns.

#### Training data: Consent and transparency

Development of machine learning systems often requires large amounts of training data on which the system can learn to recognise patterns. In line with the ethical principle of autonomy, it is key that participants should be given the option to consent. In both clinical trials and ongoing development and use of the system, patients and carers need to give their full consent for data collection. Both as an ethical commitment and in line with data protection law, consent should be freely given on an informed basis of understanding the nature of involvement and data collection and the potential consequences. The consent process for TIHM was completed according to NIHR guidelines and compliant with ethical recommendations. Participants were given information and explanations about the trial and consent was completed according to the Mental Capacity Act, under which a participant is assumed to have capacity to take a specific decision at the time in question unless demonstrably lacking that capacity. People living with dementia were asked to nominate an informal carer who would be invited to the study, providing for practical support in navigating the demands of participation. Despite these provisions for initial overt consent and ongoing support, the potential for fluctuating or declining capacity to make such decisions among people living with dementia remains, as does the more general tendency for people to become less aware of passive monitoring over time. While it might be appropriate to assume ongoing consent, any apparent dissent and particularly problems in taking part in active data collection, would signal that continuing smart care might not be appropriate.

Representativeness of training data has been identified as a key factor in developing machine learning systems that meet the ethical requirement of fairness to all.^29,30^ In smart healthcare trials, it is unlikely that it will be possible to recruit participants on a randomized or representative basis, and the potential for bias therefore needs to be kept in mind as the system develops. Reliance on participants to self-select or be referred by carers and the distinctive demographics of the local population place limits on capacity to produce a system that can be guaranteed in advance to be applicable to all people living with dementia across diversities in race, class, gender and housing situations. This concern is to some extent offset by the development of personalized thresholds for alerts^31^ but it is important in any new instance of implementation that the applicability of the system be reassessed for the new population.

#### Privacy and acceptability

Avoidance of undue intrusion on privacy is one of the core components of the ethical principle of non-maleficence. It follows that the collection of data should be proportionate to the task being undertaken and this consideration has implications for the choice of sensors to be installed in homes and the burden to be required of participants. Such considerations lead to the use of simple to install and relatively non-intrusive sensors such as smart plugs to monitor appliance usage and PIR sensors to capture movement, rather than monitoring via video camera. Such off-the-peg devices are becoming a feature in smart homes outside of the care context and hence may help to avoid the obtrusiveness and accompanying risk of stigma and loss of autonomy that Mittelstadt^22^ suggests can result when people are made visibly aware of being monitored to excess. There is a trade-off between continued consent through awareness and excessive consciousness of being watched.

A good principle for privacy protection is to minimize the extent to which any identifiable personal data is shared. This has to be balanced against the need for data from users to train the machine learning system. A centralised approach to machine learning would require user data to be sent to a hub to be used for training the system to recognize patterns. Zhao et al.^32,33^ propose a ‘federated learning model’ for in-home healthcare monitoring applications, allowing data to be kept locally by individual users and pre-processed there before sending a depersonalized aggregated set of results back to be used in improving the central model. In this way, the system can enact a principle of non-maleficence. It should be noted, however, that such measures operationalise privacy and security at the household level and do not necessarily offer people living with dementia and their carers or visitors privacy from one another. Special attention should also be given to security and privacy measures to reduce the risk of unauthorised access to the in-home stored data.

#### Performance targets and well-being

Performance for a smart healthcare system is assessed both in terms of the extent to which it meets clinical goals and in terms of the technical performance of the machine learning system. In a clinical sense, the overall goal of the system is a beneficent one: to improve care, and more specifically to lessen hospital admissions and increase the time spent living at home, assuming that this is what the person receiving care wants^28,31,34^ and thus in turn to make more efficient use of limited resources by reducing the cost of care. It can be difficult to quantify how far the system achieves these goals without long-term clinical trials and in situations where a double-blind randomised control trial is not possible. In a clinical study of the TIHM system, participants showed significant reduction in neuropsychiatric symptoms and high levels of satisfaction throughout.^11^ The example explored in this article focuses on health-related issues in dementia, such as urinary infections, agitation and falls, with a view to averting serious health issues and reducing hospitalizations. It is important to note that the criteria for success set by clinical assessments of impact and also shaped by the practicalities of the available data may not always address what people living with dementia and carers see as the most significant issues for them according to their lived experience. User-centred design techniques can help to prioritise system design according to the features most important to users.^35^

Machine learning systems do not make absolute predictions, but evaluate outcomes according to probabilities. A machine learning model will only be accepted when it reaches a level of accuracy deemed acceptable: this is often done by splitting the available training data into sets, one used for training and one used to assess performance. Predictions will not be completely accurate: it is to be expected that some alerts will be false positives, and some positive events will not generate alerts. Data collected from domestic settings is also bound to be ‘noisy’ due to malfunction of sensors, user errors and unanticipated events in the home: including the daily traffic of informal and formal carers in and out of the home that may be usual for a person living with dementia. Training the system to recognize an event such as a urine infection requires advanced techniques to bring accuracy to an acceptable level because of this noisiness of the data and because training data containing verified instances of urine infections is relatively rare.^27^ Following training of the system, a system-generated alert is produced according to a threshold set according to a human judgment about the possible consequences of a false positive or a false negative. While it is important not to miss a clinically significant event, there may be adverse consequences if too many alerts are generated, in terms of the use of clinical resources and also the well-being of patients and carers. For example, when fine-tuning the system for detecting potential urine infections based on physiological and behavioural data, it is important to set alert levels to avoid unnecessary prescribing of antibiotics.^26,27^ Setting personalized thresholds for triggering alerts based on physiological measurements proved to reduce the number of alerts to a manageable level.^31^

Above, we have detailed some of the steps that it is possible for a multi-disciplinary team to take in order to build an ethically conscious infrastructure for smart care. However, as Mittelstadt^22^ argues, there are limits to the extent to which ethics can be designed into a smart healthcare system and much is dependent on the specifics of deployment. We have described steps that can be taken in the technical design process for machine learning and the embedding of that system into a clinical context. In the next section, issues relating to the emergence of everyday ethics in the smart care setting are explored.

### Everyday ethics and smart care for dementia

In this section, attention turns to the ‘care’ component of smart care systems and to thinking about ethics in terms of caring human relations. Care for the elderly and vulnerable, including those living with dementia, exists in a liminal space between medical care and the care of kin for one another, and healthcare professionals are often involved alongside informal carers such as partners and family members. Pols^36^ suggests that we should expect ideas about what counts as good care to emerge in relations between people as they participate in caring relationships, rather than expecting to define a singular pre-existing notion of good care. Networks of care develop around the cared-for person including monitoring staff, GPs, informal carers and the technologies themselves.^36^ Monitoring technologies participate in these relations, as they afford certain activities and preclude others, as they make new forms of knowledge available and as they shift responsibilities for action on the basis of that knowledge. A form of dynamic ‘careography’^37^ emerges as the different parties navigate care decisions and their accountability for them, and we might expect that an AI system might significantly intervene in this process. Substantial existing work in the specific domain of everyday ethics in telecare for older and vulnerable patients (for example Refs. 38–41) suggests that the technology gains its ethical meaning within the everyday interactions between those involved in care. Dignity is a particular focus for concern, bearing in mind the need for compassion, preservation of autonomy and sense of self-worth and the provision of a humane and purposeful environment identified by Tranvåg et al.^16^ as vital to dignity-preserving dementia care. It is important that users feel that the implementation of smart care augments their dignity.

While the remote monitoring of smart care is aimed at enhancing the ability to treat conditions before they become serious, in doing so, it potentially extends the medicalization of care space by extending a prospective medical care further into daily experience.^42^ When implementing smart care in the context of those who are vulnerable, it is important to recognize the capacity of care recipients and to grant them such autonomy as they can retain. Living well with dementia involves participating in one’s own care decisions and in wider society.^43^ Smart care is built on the idea of maintaining independence and keeping people out of residential care for as long as possible, but it is important to recognize that this is not always the preference of the care recipient and that wishes may change over time. While ageing in place has become a widely held aspiration for care, it may not always be the desired option and quality of life, and well-being need to be taken into account.^44^ If remote care replaces in-person care, there are risks of social isolation and also a more subtle but consequential ethical risk of reducing the care relationship to that which can be sensed and measured.^22^ It is therefore relevant to ask, how the experience of being cared-for is impacted and to what extent this confers feelings of connection and autonomy on the care recipient?

Smart care systems are often promoted as offering reassurance. This framing aligns with a commonly held association of care with worry^45^ and with caring as a relation that involves both labour and emotion^46^ and can be an emotional rollercoaster.^47^ In the recent COVID-19 pandemic, the challenge of achieving care at a distance and balancing multiple concerns about the safety of those who are cared-for has increased, and we have become acutely conscious that care is more than simply practical labour. There will be emotional dimensions to the introduction of smart care systems that may simultaneously be seen as saviour and compromise solution, offering reassurance, yet not the same as warm human contact. There will be daily dilemmas to resolve around who responds to alerts and in what way, who performs the labour of maintaining sensors and taking physiological readings, alongside larger decisions around changing needs and long-term trajectories that may be prompted by the insights into the future offered by analysis of monitoring patterns. Continued research will be needed to explore how best to support healthcare professionals and informal carers to make such decisions in conjunction with the person being cared for in ways that all concerned consider to be ethical.

When navigating the territory of ethical smart care, it is important to be conscious of prevailing inequalities in understanding of the potential and the possible pitfalls of these technologies across the population: as Gran et al.^48^ argue, inequality in awareness of the operation of machine learning algorithms has the potential to constitute a new digital divide. Informed consent is at the heart of ethical healthcare, but lack of understanding can remove the possibility for consent to be fully informed and is likely to be found disproportionately in the older population also more at risk of dementia.^48^ It is likely that the healthcare professionals who have the most direct contact with those experiencing smart care will find themselves needing to educate and reassure care recipients and will themselves therefore need a good practical and ethical grasp of the system.

## Conclusion

Developing an ethical smart care system for people living at home with dementia is a complex challenge that involves intersection of different ethical frameworks and draws on multiple forms of expertise. As described above, smart healthcare systems are subject to pre-emptive ethical review that aims to identify and avoid or mitigate the worst ethical harms. There are, as described, many steps that the designers of such systems can take to embed ethical principles into the design of the infrastructure for smart care.

Table 1 summarises these design measures mapped onto the principles for ethical AI and also summarises issues relating to care ethics that cannot so easily be designed into the system. Whatever pre-emptive steps are taken, we may thus expect that there will be everyday ethical dilemmas in the implementation of smart care systems as those involved in offering and receiving care find themselves incorporating the system into their judgments and their daily routines. Healthcare professionals involved in delivery of smart care may find themselves facing challenges to their understanding of ethical practice at the same time as they are called upon to act as ambassadors for the technology, taking on a role of education and reassurance for users who may have limited technical understanding of the system. It is therefore clear that there is a need for research into the lived experience of smart care systems to be conducted alongside the technical and clinical research that enables their implementation. Such research should feed into the design process itself, into development of stronger pre-emptive ethical review for such systems and into training and support for the healthcare professionals involved in their implementation.

**Table 1.** Summary of sites of ethical concern within home-based smart care for dementia.

AI ethics	Beneficence	•System goals set according to clinical priorities •Clinical evaluation of outcomes •Appropriate personalized thresholds set for alerts
	Non-maleficence	•Privacy and security as design priorities •Data collected and stored according to informed consent and data protection legislation •Sensors chosen for acceptability
	Autonomy	•Human decision-making embedded in the system
	Fairness	•Requirement for representative training data
	Explicability	•Testing and evaluation conducted to meet standards for publication •Subject to clinical device approvals
Care ethics	Relations and locations of care	•Preservation of human contact and sense of home •Negotiating an ethical role for AI across participants in ecologies of care •Tensions between reassurance/worry •New forms of care work emerging •Implications for autonomy and dignity in care
	Decision-making	•Support for meaningful ongoing consent •Coordinating response to AI-generated risk assessments •Digital inequalities and access to care decisions
